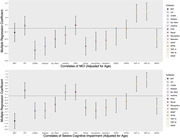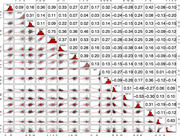# Cognitive Impairment in Firefighters and Emergency Medical Services Workers after Severe WTC Exposures

**DOI:** 10.1002/alz.089154

**Published:** 2025-01-09

**Authors:** Frank D Mann

**Affiliations:** ^1^ Stony Brook University, Stony Brook, NY USA

## Abstract

**Background:**

Members of the Fire Department of New York (FDNY) who responded to the World Trade Center (WTC) attacks that occurred on 9/11/2001 were exposed to severe trauma. The present study screened for cognitive impairment in a prospective cohort study of FDNY fire fighters and emergency personnel who reside in the greater NYC metropolitan area.

**Method:**

A large sample (n = 338) of FDNY personnel who were exposed to the WTC attacks were recruited to complete a large battery of neuropsychiatric and psychological tests. The Montreal Cognitive Assessment (MoCA) was used to screen for mild cognitive impairment (MCI) and probable dementia. Neuropsychiatric, psychological, and physical deficits were further characterized by the Short Physical Performance Battery (SPPB), Hopkins Verbal Learning Task (HVLT‐R), Boston Naming Task (BNT), Wide Range Achievement Test (WRAT‐4), Trail‐Making Task (TMT), Symbol Digit Modalities Test (SDMT), Controlled Oral Word Association (COWA), Patient Health Questionnaire (PHQ‐9), and the Cognitive Function Instrument (CFI). Diagnoses of posttraumatic stress disorder (PTSD) and major depressive disorder (MDD) were made using the Structural Clinical Interview for DSM‐5.

**Result:**

Compared to the general population, prevalence of MCI (22.92%, CI.95 = [18.42%, 27.63%]) and probable dementia (4.14%, CI.95 = [2.28%, 6.85%]) were precipitously high. Compared to unimpaired firefighters, those with MCI and probable dementia exhibited statistically significant deficits (p‐values < .05) on the HVLT‐R, BNT, WRAT‐4, TMT, SDMT, and COWA, while differences on the SPPB, PHQ‐9, and CFI were not statistically significant (p‐values > .05). Prevalence of current PTSD (18.05%, CI.95 = [14.10, 22.57] and MDD (6.51%, CI.95 = [4.12%, 9.69%] were high and moderate, respectively, but not significantly associated with MCI or probable dementia (p‐values > .05).

**Conclusion:**

There is a high prevalence of MCI and probable dementia among member of the FDNY who responded to the WTC attacks. The present study further highlights the role of extreme physical exposes in the pathogenesis of cognitive impairment and dementia. This group is a high priority for public health efforts and clinicians need to anticipate MCI and dementia in treatment planning.